# Carbon dioxide for neurogenic orthostatic hypotension in adults: a novel therapy

**DOI:** 10.1093/eurheartj/ehae653

**Published:** 2024-10-07

**Authors:** Jacquie R Baker, Shaun I Ranada, Anthony V Incognito, Robert S Sheldon, Carlos A Morillo, Richard J A Wilson, Aaron A Phillips, Satish R Raj

**Affiliations:** Department of Cardiac Sciences, Cumming School of Medicine, University of Calgary, Calgary, Alberta, Canada; Libin Cardiovascular Institute, University of Calgary, Calgary, Alberta, Canada; Department of Physiology and Pharmacology, Cumming School of Medicine, University of Calgary, Calgary, Alberta, Canada; Hotchkiss Brain Institute, University of Calgary, Calgary, Alberta, Canada; Department of Cardiac Sciences, Cumming School of Medicine, University of Calgary, Calgary, Alberta, Canada; Libin Cardiovascular Institute, University of Calgary, Calgary, Alberta, Canada; Department of Physiology and Pharmacology, Cumming School of Medicine, University of Calgary, Calgary, Alberta, Canada; Hotchkiss Brain Institute, University of Calgary, Calgary, Alberta, Canada; Department of Cardiac Sciences, Cumming School of Medicine, University of Calgary, Calgary, Alberta, Canada; Libin Cardiovascular Institute, University of Calgary, Calgary, Alberta, Canada; Department of Cardiac Sciences, Cumming School of Medicine, University of Calgary, Calgary, Alberta, Canada; Libin Cardiovascular Institute, University of Calgary, Calgary, Alberta, Canada; Department of Physiology and Pharmacology, Cumming School of Medicine, University of Calgary, Calgary, Alberta, Canada; Hotchkiss Brain Institute, University of Calgary, Calgary, Alberta, Canada; Alberta Children’s Hospital Research Institute for Child and Maternal Health, University of Calgary, Calgary, Alberta, Canada; Department of Cardiac Sciences, Cumming School of Medicine, University of Calgary, Calgary, Alberta, Canada; Libin Cardiovascular Institute, University of Calgary, Calgary, Alberta, Canada; Department of Physiology and Pharmacology, Cumming School of Medicine, University of Calgary, Calgary, Alberta, Canada; Hotchkiss Brain Institute, University of Calgary, Calgary, Alberta, Canada; Department of Cardiac Sciences, Cumming School of Medicine, University of Calgary, Calgary, Alberta, Canada; Libin Cardiovascular Institute, University of Calgary, Calgary, Alberta, Canada

**Keywords:** Neurogenic orthostatic hypotension, Carbon dioxide, Blood pressure, Blood pressure therapy

## Introduction

Neurogenic orthostatic hypotension (nOH) is a hallmark feature of autonomic nervous system failure.^1^ On standing, people with nOH experience large reductions in blood pressure (≥20/10 mmHg) and debilitating symptoms (e.g. light-headedness, blurred vision, and syncope).^1^ Current blood pressure therapies have limited efficacy and potentially serious side effects, including supine hypertension,^2^ creating a strong clinical need for new therapeutic approaches.

Previous studies examining the effects of hypocapnia [i.e. low arterial carbon dioxide (CO_2_)] on blood pressure have shown that hyperventilation-induced hypocapnia decreases supine blood pressure in nOH patients and can be readily prevented with exogenous CO_2_.^3,4^ Whether exogenous CO_2_ can prevent OH in these patients is unknown. In this proof-of-concept study, we tested the hypothesis that increased inspired CO_2_ increases standing blood pressure in patients with nOH.

## Methods

We performed a randomized, unblinded study in male and female nOH patients.^1^ All participants provided written informed consent prior to participating. Respired end-tidal CO_2_ (ETCO_2_) and ETO_2_ were controlled using prospective gas targeting. Breath-by-breath ETO_2_, ETCO_2_, and ventilation were measured. Participants were instrumented with a non-invasive beat-to-beat finger blood pressure cuff (Finapres Nova, FMS, the Netherlands), and a three-lead electrocardiogram. Beat-to-beat blood pressure was analysed using Modelflow Waveform Analysis to obtain estimates of stroke volume, cardiac output, and systemic vascular resistance.

### Experimental paradigm

Participants completed three sit-to-stand tests breathing: (i) 0 mmHg CO_2_ relative to baseline (0CO_2_: ETCO_2_ clamped at baseline), (ii) +5 mmHg CO_2_ relative to baseline (+5CO_2_), and (iii) +10 mmHg CO_2_ relative to baseline (+10CO_2_). Interventions were randomized using a computer-generated randomizer. Each condition consisted of a minimum 5-min seated baseline followed by a 5-min stand (as tolerated). Stands were separated by a seated 10-min rest to allow cardiorespiratory parameters to normalize. Data were acquired at a sampling frequency of 1000 Hz (WinDaq, DATAQ Corp) for off-line analysis (LabChart Pro 8, ADInstruments, New Zealand). Average baseline cardiorespiratory data were calculated over the final 2 min of seated baseline preceding each stand. Standing cardiorespiratory data were averaged across each minute. Delta (Δ) values were calculated as the average final minute of stand-average baseline.

### Statistical analysis

Data are presented as mean ± standard deviation unless indicated otherwise. A Shapiro–Wilk test was used to determine normality. The primary endpoint was the elevation in systolic blood pressure (SBP) in response to CO_2_ (0CO_2_ vs. +10CO_2_). Using a paired *t*-test and our own preliminary data, we estimated 15 patients would have 80% power to detect a difference with an *α* = 0.05. Secondary analyses compared cardiorespiratory changes across CO_2_ condition using repeated measures analysis of variance with Bonferroni corrections for multiple comparisons or a mixed-effects model if values were missing. Two-factor mixed-effects models with Bonferroni corrections were used to evaluate interactions between CO_2_ and time. A proportion of patients meeting OH criteria between 0CO_2_ and +10CO_2_ were compared using a paired McNemar test. Statistical significance was set at *P* < .05. Statistical analyses were performed using GraphPad Prism (v.9.4.1, GraphPad, USA). Figures were created using GraphPad Prism, and Adobe Illustrator (Adobe Systems Inc., USA).

## Results

### Cardiorespiratory responses to increased inspired CO_2_

**Figure 1:**
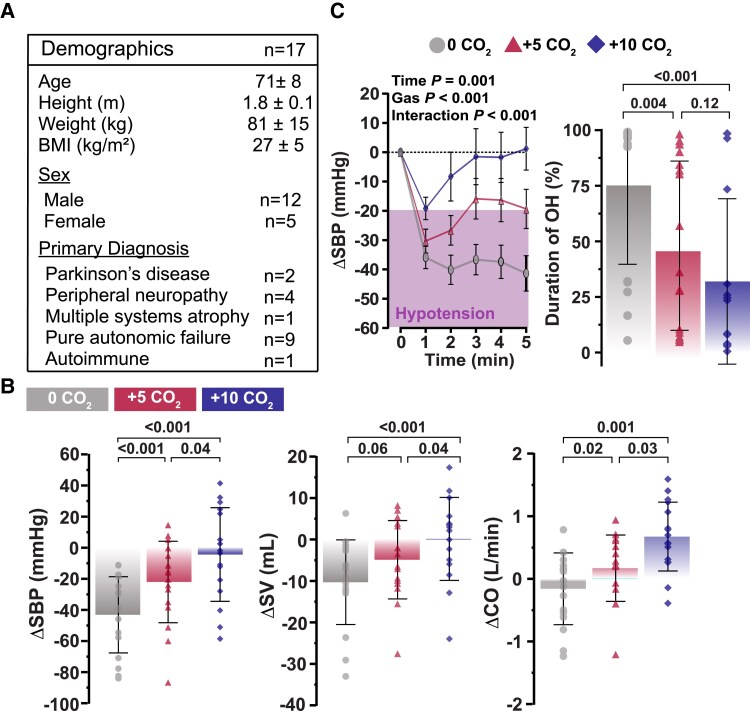
Cardiorespiratory changes associated with increased inspired CO_2_ in patients with neurogenic orthostatic hypotension. (*A*) In 17 patients with neurogenic orthostatic hypotension, (*B*) systolic blood pressure (*P* < .001), stroke volume (*P* < .001), and cardiac output (*P* > .001) each showed a robust dose-dependent increase across each CO_2_ condition. (*C*) CO_2_ increased systolic blood pressure as a function of time (*P* < .001) and reduced the duration of orthostatic hypotension (*P* < .001). A mixed-effects model with a Bonferroni correction for multiple comparisons was used to compare the changes in cardiorespiratory parameters and to compare duration of OH between CO_2_ conditions. A two-factor mixed-effects model with Bonferroni corrections were used to evaluate CO_2_ condition and time interactions. Circles = 0 CO_2_, Triangles = +5 CO_2_, and Diamonds = +10 CO_2_. Purple shading represents threshold for orthostatic hypotension (systolic drop ≥ 20 mmHg). Data for minute-by-minute changes are presented as mean ± SEM. BMI, body mass index; CO, cardiac output; CO_2_, carbon dioxide; OH, orthostatic hypotension; SBP, systolic blood pressure; SV, stroke volume

Seventeen patients participated in this study (*Figure 1A*). Compared with 0CO_2_, +10CO_2_ increased SBP (0CO_2_: −44 ± 25 mmHg; +10CO_2_: −4 ± 30 mmHg; *P* < .001). Systolic blood pressure also showed a robust dose-dependent increase across each CO_2_ condition (0CO_2_: 100 ± 23 mmHg; +5CO_2_: 123 ± 21 mmHg; +10CO_2_: 135 ± 29 mmHg; *P* < .001) and as a function of time (*Figure 1B and C*). Changes in SBP were predominantly facilitated by dose-dependent increases in stroke volume and cardiac output (*Figure 1B*). In contrast, neither the change in heart rate (0CO_2_: 10 ± 5 b.p.m.; +5CO_2_: 11 ± 5 b.p.m.; +10CO_2_: 13 ± 7 b.p.m.; *P* = .16) nor systemic vascular resistance (0CO_2_: −703 ± 639 dynes/s/cm^5^; +5CO_2_: −602 ± 604 dynes/s/cm^5^; +10CO_2_: −567 ± 551 dynes/s/cm^5^; *P* = .61) were different across conditions.

### Orthostatic hypotension criteria

At 0CO_2_, 12/17 (71%) patients met the SBP criteria for OH.^1^ During +5CO_2_, 8/17 (47%) patients met OH criteria, while only 4/16 (25%) met OH criteria during +10CO_2_. Further, +10CO_2_ reduced the proportion of patients meeting OH criteria compared with 0CO_2_ (*P* = .02). Importantly, increased inspired CO_2_ reduced the duration of OH while patients were standing (*Figure 1C*).

## Discussion

Here, we show for the first time that increased inspired CO_2_ powerfully elevates *standing* blood pressure in patients with nOH and reduces the duration of OH. Rather than through changes in heart rate or systematic vascular resistance, this response was primarily driven by an increase in stroke volume and cardiac output. This haemodynamic profile strongly suggests that inspired CO_2_ increases blood pressure via enhanced cardiac venous return. While the precise mechanisms remain unknown, residual sympathetic nerve activity, which has been postulated in the pathogenesis of supine hypertension in nOH patients;^5^ a direct constrictor effect of CO_2_ on blood vessels;^3,4^ and increased ventilation/ventilatory effort to produce a negative inspiratory pressure^6^ are all potential mechanisms underlying the observed pressor response.

As OH is associated with an increased risk of injurious falls and OH-related hospitalizations,^7–10^ identifying an effective pressor therapy could have a broader clinical benefits beyond mere blood pressure elevation including reduced fall risk, OH-related hospitalizations, and associated healthcare costs. Other acute benefits could include improved orthostatic tolerance and enhanced ability to perform daily activities.

Despite these findings, the following limitations warrant consideration, including the use of a sit-to-stand manoeuvre, which may elicit smaller haemodynamic changes compared with a standard head-up tilt or supine-to-stand test; the short exposure to CO_2_, which precludes us from understanding the long-term implications of this intervention; the relatively smaller sample size; and the absence of detailed mechanistic insights into CO_2_-mediated pressor effects. Lastly, we evaluated patients with *neurogenic* OH and, therefore, cannot comment on the efficacy of CO_2_ in other forms of OH. However, the observed dose–response suggests that, with appropriate titration, CO_2_ therapy could potentially benefit patients with mild, moderate, or severe OH. Addressing these limitations in future research could provide a more comprehensive understanding of the therapeutic potential of CO_2_ in nOH management.

## Conclusions

Increased inspired CO_2_ elevates standing blood pressure in patients with nOH and reduces the duration of OH. These findings support further exploration of CO_2_ delivery interventions as an acute blood pressure therapy in nOH, potentially improving orthostatic tolerance and preventing syncope, traumatic falls, and, in turn, OH-related hospitalizations.

## Declarations

### Data Availability

S.R.R. takes responsibility for the integrity of the data and the accuracy of the data analysis. Please contact the corresponding author to discuss access to the data used in these analyses.

### Pre-registered Clinical Trial Number

NCT05295810.
